# ICD-11 and *DSM*-5-TR prolonged grief symptoms and quality of life: A criterion validity test

**DOI:** 10.1177/00048674241249601

**Published:** 2024-05-06

**Authors:** Maarten C Eisma, Lara O Schmitt

**Affiliations:** Department of Clinical Psychology and Experimental Psychopathology, University of Groningen, Groningen, The Netherlands

**Keywords:** Prolonged grief disorder, complicated grief, concurrent validity, predictive validity, well-being

## Abstract

**Objective:**

Two similar but distinct versions of prolonged grief disorder (PGD) have recently been included in the International Classification of Diseases eleventh edition (ICD-11) and the *Diagnostic and Statistical Manual of Mental Disorders* – fifth edition, Text-Revision (*DSM*-5-TR). This study provides a criterion validity test of both new criteria sets of PGD, by examining concurrent and longitudinal associations of ICD-11 and *DSM*-5-TR prolonged grief symptoms with quality of life (QOL).

**Methods:**

Bereaved adults completed a survey assessing ICD-11 and *DSM*-5-TR prolonged grief symptoms, depressive symptoms, insomnia symptoms and QOL at baseline and 6-month follow-up.

**Results:**

Both ICD-11 and *DSM*-5-TR prolonged grief symptoms related negatively to QOL concurrently, while controlling for insomnia and depressive symptoms. ICD-11 prolonged grief symptoms, but not *DSM*-5-TR prolonged grief symptoms, predicted QOL at 6-month follow-up, while controlling for baseline QOL and insomnia and depression symptoms.

**Conclusions:**

Results provide consistent evidence for the criterion validity of ICD-11 PGD, but mixed evidence for the criterion validity of *DSM*-5-TR PGD. Study results can help guide attempts to optimize and harmonize future PGD criteria.

## Introduction

Over the past decades, there has been increasing recognition that a minority of bereaved individuals experiences severe, persistent and disabling grief, currently most commonly termed prolonged grief. In 2018, a diagnosis characterized by such grief responses, prolonged grief disorder (PGD) was added to the International Classification of Diseases eleventh edition (ICD-11, World Health Organization, 2022). In 2022, a similar but distinct diagnosis, also termed PGD was added to the *Diagnostic and Statistical Manual of Mental Disorders* - fifth edition, text revision (*DSM*-5-TR, American Psychiatric Association, 2022). Across diagnostic systems, core symptoms include severe and persistent yearning for the deceased and cognitive preoccupation with the deceased. However, the number and content of secondary symptoms, timing criteria and diagnostic algorithms of ICD-11 PGD and *DSM*-5-TR PGD differ (for an overview: Eisma, 2023).

Moreover, since both criteria sets were new when first introduced, their symptom count, content and diagnostic algorithms do not correspond completely with prior proposed grief disorders, such as complicated grief (Shear et al., 2011), persistent complex bereavement disorder (*DSM*-5, American Psychiatric Association, 2013) and earlier proposals for PGD (e.g. Prigerson et al., 2009). A content overlap analysis showed only moderate content overlap between past and current criteria sets for pathological grief (Eisma et al., 2022). Frequently used measures to assess pathological grief responses, such as (versions of) the Inventory of Complicated Grief (ICG: Prigerson et al., 1995) and the Prolonged Grief scale 13 (PG-13: Prigerson et al., 2009), do not comprehensively assess PGD per ICD-11 and *DSM*-5-TR (Lenferink et al., 2022; Treml et al., 2020). A major challenge originating from this inconsistency is that we cannot assume that past research concerning the validity of grief disorders generalizes to the most recent versions of PGD (Eisma, 2023). It is therefore imperative to examine key aspects of the validity of these new disorders using measures that comprehensively assess symptoms of PGD per ICD-11 and *DSM*-5-TR, such as the recently developed Traumatic Grief Inventory Self Report Plus (TGI-SR+, Lenferink et al., 2022). The present study will shed light on the criterion validity of the newest versions of PGD by examining the concurrent and longitudinal associations of ICD-11 and *DSM*-5-TR prolonged grief symptoms with quality of life (QOL). Thereby, we will evaluate evidence for concurrent validity (i.e. the extent to which prolonged grief symptoms relate to a relevant criterion concurrently) as well as predictive validity (i.e. the extent to which prolonged grief symptoms predict a relevant criterion longitudinally) for these different symptom sets.

The World Health Organization (2022) defines QOL as *an individual’s perception of their position in life in the context of the culture and value systems in which they live and in relation to their goals, expectations, standards and concerns.* QOL is often understood to be a multidimensional construct reflective of general well-being, encompassing experienced physical health, mental health, social relationships and environmental health (Schmidt et al., 2006). There are several reasons why QOL can be regarded as a suitable criterion for PGD. First, severe grief reactions are associated with decrements in physical health, such as cardiovascular health problems, immunological deficiencies and sleep disturbances (e.g. Brown et al., 2022; Knowles et al., 2019; Lancel et al., 2020; Palitsky et al., 2023; Stroebe et al., 2007). Second, the functional impairment criterion of PGD holds that the disorder must cause clinically significant distress or impairment in social, occupational or other important areas of functioning (*DSM*-5-TR, American Psychiatric Association, 2022), which would likely be reflected in reduced QOL. Third, multiple past studies, using older pathological grief conceptualizations and measures, have demonstrated negative concurrent and longitudinal (zero-order) associations between pathological grief symptoms and QOL (e.g. Boelen and Prigerson, 2007; Maccallum and Bryant, 2020; Silverman et al., 2000; Tsai et al., 2020).

In the current study, we examined the concurrent and longitudinal associations between prolonged grief symptoms per ICD-11 and *DSM*-5-TR and QOL to shed light on the criterion validity of the newest PGD criteria sets. In order to conduct a stringent validity test, we controlled our analyses for depression and insomnia symptoms, as these conditions show strong associations with prolonged grief (Komischke-Konnerup et al., 2021; Lancel et al., 2020). In addition, we controlled our longitudinal analyses for baseline QOL, to establish whether prolonged grief symptoms per ICD-11 and *DSM*-5-TR could predict residual changes in QOL.

## Method

### Procedure

The Ethical Committee Psychology of the University of Groningen approved this General Data Protection Regulation compliant study (registration number: PSY-1819 S-0173). Data were collected from May 2019 until September 2021 as part of a longitudinal survey study on psychosocial adaptation on bereavement. Participants were recruited through advertisements on Google AdWords and via a website containing a grief self-test (www.psyned.nl). The eligibility criteria were (1) Dutch proficiency, (2) having experienced the death of a partner, family member or friend in the past 5 years (but we decided to retain data of 25 participants not strictly meeting this timing criterion to retain optimal power) and (3) being at least 18 years old.

Participants read an information page explaining the aims and procedure of the study and ethical issues (e.g. anonymity, voluntariness of participation). After online informed consent was given, a series of questionnaires were presented through Qualtrics, a secured survey program. Completion of the survey took approximately 30 minutes. A total of 820 participants completed the baseline survey (T0). Three people experiencing pet loss were excluded. The remaining 817 were asked if they would be willing to participate in a longitudinal study at the end of the questionnaire. Those interested (*N* = 561), received an email with a link to the follow-up survey 6 months (T1) and 12 months (T2) after the initial survey. Two reminders were sent, 2 and 3 weeks after the initial message, following each invitation message. For the current paper, data from T1 and T2 were used.

**Figure 1.**
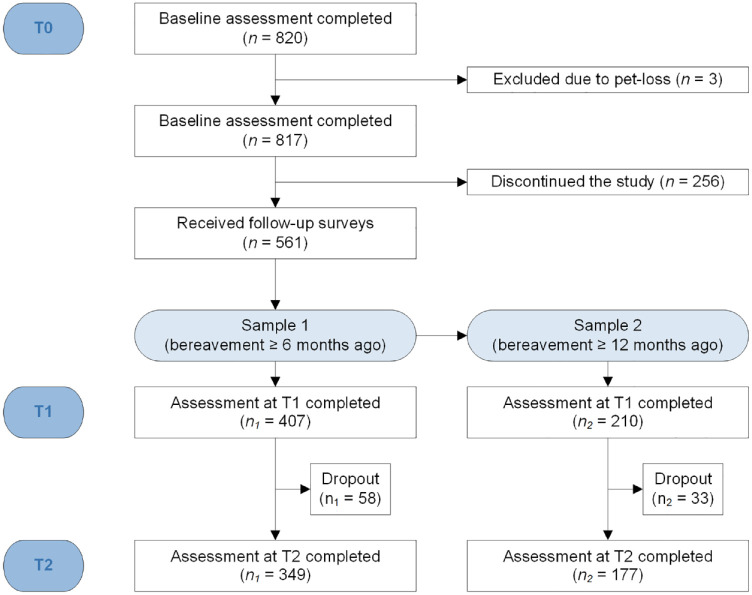
Participant flowchart.

Since the aim of the study was to examine the criterion validity of ICD-11 and *DSM*-5-TR, we selected two samples from the original dataset. Sample 1 consisted of all participants in the original study who completed the T1 survey. All these participants had experienced bereavement at least 6 months ago (i.e. time criterion for ICD-11 PGD) at T1 (*N* = 407). Sample 1 was used for the analyses on ICD-11 prolonged grief symptoms. Sample 2 consisted of a selected subsample of participants from the original dataset who experienced bereavement at least 12 months ago (i.e. time criterion for *DSM*-5-TR PGD) at T1 (*N* = 210). Sample 2 was used for the analyses on *DSM*-5-TR prolonged grief symptoms. See Figure 1 for a participant flowchart.

### Materials

#### Sociodemographic and loss-related characteristics

Sociodemographic characteristics (i.e. age, sex, educational level), as well as characteristics of the loss and the deceased (i.e. relationship to and sex of deceased, cause of death and death expectedness) were assessed with a self-constructed questionnaire.

#### Prolonged grief symptoms

To assess prolonged grief symptoms, the Traumatic Grief Inventory – Self Report Plus (TGI-SR+; Lenferink et al., 2022) was used. The TGI-SR+ consists of 22 items which are administered on a 5-point-Likert-type scale ranging from 1 (*never*) to 5 (*always*). Twelve items correspond to the ICD-11 criteria of PGD, which were summed to indicate ICD-11 prolonged grief symptoms. The TGI-SR+ ICD-11 items showed excellent internal consistency (T1: α = 0.90; T2: α = 0.90). A cut-off of ⩾40 (liberal scoring rule: one additional symptom) or ⩾41 (conservative scoring rule: ⩾5 additional symptoms) has been established for probable ICD-11 PGD. Similarly, 10 items that correspond to the *DSM*-5-TR criteria of PGD were summed to represent *DSM*-5-TR prolonged grief symptoms. The internal consistency of the TGI-SR+ *DSM*-5-TR items was good to excellent (T1: α = 0.89; T2: α = 0.90). A cut-off of ⩾33 has been established for probable *DSM*-5-TR PGD.

#### Quality of life

The total score of the Dutch version of the European Health Interview Survey – QOL (EUROHIS-QOL; Schmidt et al., 2006), a brief, globally used measure covering multiple facets of well-being, was used to assess QOL. Eight items, scored on a 5-point Likert-type scale ranging from 1 (*not at all*) to 5 (*completely*), covered with two items each psychosocial, physical, social and environmental QOL. In our study, the scale showed good internal consistency (T1: α = 0.86; T2: α = 0.85).

#### Depressive symptoms

Depressive symptoms were assessed with the Dutch 16-item Quick Inventory of Depressive Symptomology (QIDS; Rush et al., 2003; Dutch version: Lako et al., 2014). Sixteen items measured symptoms of mood, appetite, weight, psychomotor problems and sleep problems during the past week. The items were scored on a 4-point scale ranging from 0 to 3 (varying anchors). The QIDS showed good reliability (T1: α = 0.81; T2: α = 0.80).

#### Insomnia symptoms

The Insomnia Severity Index (ISI; Morin, 1993) was used to asses insomnia symptoms. On seven items, participants indicated their sleep symptoms for the past 2 weeks. Items were scored on a 5-point Likert-type scale from 0 to 4 with varying anchors. In the current study, internal consistency was good (T1: α = 0.87; T2: α = 0.86).

### Statistical analyses

For the analyses, IBM SPSS Statistic (Version 26, for IOS) was used. For all analyses, a two-sided significance level of *p* < 0.05 was used. All analyses mentioned below were run separately for ICD-11 prolonged grief symptoms (Sample 1: *N* = 407) and *DSM*-5-TR prolonged grief symptoms (Sample 2: *N* = 210).

First, a dropout analysis was conducted to compare possible differences between participants who completed T1 and T2, and those who dropped out after T1. For categorical variables (i.e. sex, education level, relationship with deceased, cause of death, expectedness of death) a Chi-square test was used. To assess differences in prolonged grief symptoms, age and time since loss between dropout and completers’ independent sample *t*-tests were conducted. Assumptions were checked and nonparametric alternative analyses were used in case assumptions were violated. Second, for exploratory purposes, zero-order correlations were calculated between T1 prolonged grief symptoms and T1 QOL, T1 prolonged grief symptoms and T2 QOL, and T2 prolonged grief symptoms and T2 QOL.

For the main analyses, three hierarchical multiple regressions predicting QOL from prolonged grief symptoms were conducted.

In the first multiple regression analysis, T1 prolonged grief symptoms predicted T1 QOL, while controlling for depressive and insomnia symptoms at T1. Thus, T1 depressive and insomnia symptoms were added in the first step of the model and T1 prolonged grief symptoms in the second step to predict T1 QOL.

In the second multiple regression analysis, T1 prolonged grief symptoms predicted T2 QOL, while controlling for T1 QOL. In the first step, T1 QOL was added to the model as a predictor of T2 QOL. In the second step, we added T1 prolonged grief symptoms to the model.

Lastly, in a third multiple regression analysis, T1 prolonged grief symptoms predicted T2 QOL, while controlling for T1 QOL as well as T1 depressive and insomnia symptoms. Hence, T1 QOL was added in the first step, T1 depressive and insomnia symptoms were added in the second step and we added T1 prolonged grief symptoms in the third step to predict T2 QOL.

The model assumptions of normality, linearity and homoscedasticity were visually inspected by examining scatterplots, residual plots and P–P plots. Multicollinearity was assessed by calculating tolerance values and variance inflation factors (VIFs). Outliers were examined by calculating Cook’s distances.

## Results

### Preliminary analyses

#### Sample characteristics

##### Sample 1

In Sample 1 (selected for analyses on ICD-11 prolonged grief symptoms), the 407 participants (*M*_age_ = 53.16, *SD* = 12.73, range = 19–84) were predominantly women (89%) who had completed college or university (58%). On average, the loss occurred 20 months ago (*M* = 19.48, *SD* = 16.80, range: 7–67). Most participants had lost a partner (47%), or a parent (31%), due to natural causes (86%). Half of the participants experienced the loss as unexpected (50%).

##### Sample 2

Sociodemographic and loss-related characteristics of 210 participants in Sample 2, selected for analyses on *DSM*-5-TR prolonged grief symptoms, were highly similar to the characteristics of Sample 1; see Table 1.

**Table 1.** Baseline sample characteristics for Samples 1 and 2.

	Sample 1 (*N* = 407) (ICD-11)	Sample 2 (*N* = 210) (*DSM*-5-TR)
Sex, *n* (%)		
Woman	360 (89)	186 (89)
Age in years, M (SD)	53.16 (12.73)	53.66 (13.47)
Level of education^a^, *n* (%)		
Lower education	172 (42)	89 (42)
Higher education	235 (58)	121 (58)
The deceased was, *n* (%)		
Partner	193 (47)	112 (53)
Parent	125 (31)	47 (22)
Sibling	23 (6)	14 (7)
Child	49 (12)	27 (13)
Other persons	17 (4)	10 (5)
Time since the loss in months, M (SD)	19.48 (16.80)	30.05 (17.75)
Cause of death^b^, *n* (%)		
Nonviolent	349 (86)	171 (82)
Violent	58 (14)	39 (19)
Sex of the deceased, *n* (%)		
Man	274 (67)	155 (74)
The loss was, *n* (%)		
Expected	136 (33)	68 (32)
Unexpected	204 (50)	111 (53)
Both or neither	67 (17)	31 (15)

ICD-11: International Classification of Diseases eleventh edition; *DSM*-5-TR: *Diagnostic and Statistical Manual of Mental Disorders* - fifth edition, text revision.

a Higher education = college and university, lower education = education levels lower than college or university.

b Nonviolent loss = natural deaths and coronavirus deaths, violent loss = accident or suicide.

##### Comparisons of Sample 1 and Sample 2

We could not conduct direct comparisons of both samples because they partially overlapped. Instead, we compared participant characteristics of Sample 2 against people not included in Sample 2 (but included in Sample 1). Two differences emerged: people in Sample 2 were more likely to have experienced the death of a man (vs woman) and more likely to have experienced a violent loss (vs nonviolent loss); see Supplemental Table A.

#### Dropout analyses

##### Sample 1

For Sample 1, 407 participants completed the T1 survey. Of these participants, 58 (14%) did not complete the T2 survey. For sex, educational level, expectedness of the death, time since loss and prolonged grief symptoms per ICD-11, no significant difference between completers and dropouts was detected. However, people who dropped out were more likely to have experienced an unnatural death, χ^2^ (1, *N*_dropout_ = 14 (24%), *N*_completers_ = 44 (13%)) = 5.41, *p* = 0.02, and were younger, *M*_dropout_ = 48.55, *M*_completers_ = 53.92; *U* = 7530.50, *p* = 0.002. In addition, participants who lost a partner were more likely to complete the study, χ^2^ (1, *N*_dropout_ = 20 (34%), *N*_completers_ = 173 (49%)) = 4.54, *p* = 0.033.

##### Sample 2

For Sample 2, 210 participants completed the T1 survey. Of these participants, 33 (16%) did not complete the T2 survey. Dropout patterns were similar. For sex, educational level, expectedness of the death, time since loss and prolonged grief symptoms per *DSM*-5-TR, no significant difference between completers and dropouts was detected. However, people who dropped out were more likely to have experienced an unnatural death, χ^2^ (1, *N*_dropout_ = 11 (33%), *N*_completers_ = 28 (16%)) = 5.64, *p* = 0.018, and were younger *M*_dropout_ = 49.27, *M*_completers_ = 54.47; *U* = 2171.50, *p* = 0.019. In addition, participants who lost a partner were more likely to complete the study, χ^2^ (1, *N*_dropout_ = 11 (33%), *N*_completers_ = 101 (57%)) = 6.29, *p* = 0.012.

#### Descriptive statistics and zero-order correlations

In Samples 1 and 2, symptoms of prolonged grief, depression and insomnia ranged from clinical to nonclinical and reduced over time (cf. de Lang et al., 2023), whereas QOL remained relatively stable (see Tables 2 and 3). Moreover, in both samples, symptom measures were positively correlated with each other, whereas QOL was consistently negatively correlated with symptoms of prolonged grief, depression and insomnia.

**Table 2.** Descriptive statistics and Pearson’s correlations for main study variables in Sample 1.

	*N*	*M* (*SD*)	1.	2.	3.	4.	5.	6.	7.	8.	9.
1. ICD-11 PGS T1	407	34.69 (9.04)									
2. ICD-11 PGS T2	355	32.45 (9.07)	0.82								
3. *DSM*-5-TR PGS T1	407	30.68 (7.79)	0.96	0.78							
4. *DSM*-5-TR PGS T2	355	28.82 (7.87)	0.78	0.95	0.80						
5. QOL T1	407	27.70 (5.71)	−0.61	−0.52	−0.61	−0.52					
6. QOL T2	349	28.31 (5.25)	−0.56	−0.59	−0.56	−0.59	0.78				
7. Depressive symptoms T1	407	9.48 (5.16)	0.68	0.58	0.69	0.57	−0.78	−0.67			
8. Depressive symptoms T2	351	8.62 (4.93)	0.61	0.66	0.60	0.65	−0.67	−0.74	0.77		
9. Insomnia symptoms T1	407	8.57 (5.43)	0.44	0.43	0.43	0.42	−0.47	−0.42	0.59	0.53	
10. Insomnia symptoms T2	349	7.95 (5.10)	0.40	0.48	0.37	0.46	−0.44	−0.49	0.52	0.62	0.76

PGS: prolonged grief symptoms; QOL: quality of life; ICD-11: International Classification of Diseases eleventh edition; *DSM*-5-TR: *Diagnostic and Statistical Manual of Mental Disorders* - fifth edition, text revision.

All correlations are significant (all *p’s* < 0.01).

**Table 3.** Descriptive statistics and Pearson’s correlations for main study variables in Sample 2.

	*N*	*M* (*SD*)	1.	2.	3.	4.	5.	6.	7.	8.	9.
1. ICD-11 PGS T1	210	33.38 (9.20)									
2. ICD-11 PGS T2	182	31.76 (8.78)	0.83								
3. *DSM*-5-TR PGS T1	210	29.49 (7.90)	0.96	0.79							
4. *DSM*-5-TR PGS T2	182	28.16 (7.43)	0.79	0.95	0.80						
5. QOL T1	210	27.75 (5.85)	−0.61	−0.52	−0.60	−0.50					
6. QOL T2	177	28.34 (5.10)	−0.54	−0.58	−0.50	−0.56	0.78				
7. Depressive symptoms T1	210	9.20 (5.11)	0.65	0.59	0.64	0.57	−0.76	−0.64			
8. Depressive symptoms T2	179	8.40 (4.88)	0.60	0.69	0.57	0.67	−0.66	−0.73	0.76		
9. Insomnia symptoms T1	210	8.21 (5.30)	0.39	0.41	0.35	0.39	−0.44	−0.36	0.56	0.49	
10. Insomnia symptoms T2	177	8.10 (5.26)	0.35	0.47	0.31	0.44	−0.44	−0.49	0.51	0.64	0.77

PGS: prolonged grief symptoms; QOL: quality of life; ICD-11: International Classification of Diseases eleventh edition; *DSM*-5-TR: *Diagnostic and Statistical Manual of Mental Disorders* - fifth edition, text revision.

All correlations are significant (all *p’s* < 0.01).

#### Assumption checks

For the planned hierarchical regressions, we checked the model assumptions of normality, linearity and homoscedasticity as well as (lack of) multicollinearity. We detected no violations of assumptions.

### Main analyses

#### Associations of ICD-11 prolonged grief symptoms and quality of life (Sample 1)

Three hierarchical regressions were performed to examine the cross-sectional and longitudinal association between ICD-11 prolonged grief symptoms and QOL (see Table 4).

**Table 4.** Multiple regression analyses on associations of ICD-11 and *DSM*-5-TR prolonged grief symptoms and quality of life.

	Sample 1 (ICD-11)			Sample 2 (*DSM*-5-TR)		
DV: T1 QOL	Δ*F*	Δ*R*^2^	*b* (95% CI)	Δ*F*	Δ*R*^2^	*b* (95% CI)
Step 1	302.91**	0.60		143.79**	0.58	
T1 Depressive symptoms			−0.75**(−0.85, −0.64)			−0.73**(−0.87, −0.58)
T1 Insomnia symptoms			0.00(−0.08, 0.08)			−0.02 (−0.13, 0.10)
Step 2	11.90**	0.01		10.36**	0.02	
T1 Prolonged grief symptoms			−0.09**(−0.15, −0.04)			−0.14**(−0.22, −0.05)
DV: T2 QOL	Δ*F*	Δ*R*^2^	*b* (95% CI)	Δ*F*	Δ*R*^2^	*b* (95% CI)
Step 1	554.95**	0.62		276.16**	0.62	
T1 QOL			0.64(0.57, 0.72)			0.65**(0.55, 0.75)
Step 2	11.57**	0.01		0.91	0.00	
T1 Prolonged grief symptoms			−0.08(−0.13, −0.03)			−0.04(−0.11, 0.04)
DV: T2 QOL	Δ*F*	Δ*R*^2^	*b* (95% CI)	Δ*F*	Δ*R*^2^	*b* (95% CI)
Step 1	554.95**	0.62		276.16**	0.61	
T1 QOL			0.59**(0.49, 0.68)			0.60**(0.47, 0.73)
Step 2	4.84**	0.01		1.23	0.01	
T1 Depressive symptoms			−0.09(−0.22, 0.03)			−0.11(−0.27, 0.06)
T1 Insomnia symptoms			−0.02(−0.10, 0.06)			0.01(−0.10, 0.12)
Step 3	5.30*	0.01		0.180	0.00	
T1 Prolonged grief symptoms			−0.06*(−0.11, −0.01)			−0.02(−0.10, 0.06)

ICD-11: International Classification of Diseases eleventh edition; *DSM*-5-TR: *Diagnostic and Statistical Manual of Mental Disorders* - fifth edition, text revision; DV: dependent variable; 95% CI: 95% Confidence Interval.

* *p* < 0.05; **p < 0.01.

The first hierarchical regression explained 61% of variance in T1 QOL, *F* (3, 403) = 211.36, *p* < 0.001. In Step 1, T1 depressive and insomnia symptoms were entered to the model explaining 60% of the variance in T1 QOL. In Step 2, T1 ICD-11 prolonged grief symptoms was added to the model, explaining 1% additional variance in T1 QOL.

The second hierarchical regression explained 63% of variance in T2 QOL, *F* (2, 346) = 291.72, *p* < 0.001. In Step 1, T1 QOL was entered to the model explaining 62% of variance in T2 QOL. In Step 2, T1 ICD-11 prolonged grief symptoms were added to the model, explaining 1% additional variance in T2 QOL.

The third hierarchical regression explained 64% of variance in T2 QOL, *F* (4, 344) = 147.35, *p* < 0.001. In Step 1, T1 QOL was entered to the model explaining 62% of variance in T2 QOL. In Step 2, T1 depressive and insomnia symptoms were entered to the model explaining 1% additional variance in T2 QOL. In Step 3, T1 ICD-11 prolonged grief symptoms were added to the model explaining 1% additional variance in T2 QOL.

#### Associations of *DSM*-5-TR prolonged grief symptoms and quality of life (Sample 2)

Three hierarchical regressions were run to examine cross-sectional and longitudinal associations between *DSM*-5-TR prolonged grief symptoms and QOL (see Table 4).

The first hierarchical regression explained 60% of variance in T1 QOL, *F* (3, 206) = 103.64, *p* < 0.001. In Step 1, T1 depressive and insomnia symptoms were entered to the model explaining 58% of the variance in T1 QOL. In Step 2, T1 *DSM*-5-TR prolonged grief symptoms were added to the model, explaining 2% additional variance in T1 QOL.

The second hierarchical regression explained 61% of variance in T2 QOL, *F* (2, 174) = 138.47, *p* < 0.001. In Step 1, T1 QOL was entered to the model explaining 61% of the variance in T2 QOL. In Step 2, T1 *DSM*-5-TR prolonged grief symptoms were added to the model, explaining 0% additional variance in T2 QOL.

The third hierarchical regression explained 62% of variance in T2 QOL, *F* (4, 172) = 69.55, *p* < 0.001. In Step 1, T1 QOL was entered to the model explaining 61% of variance in T2 QOL. In Step 2, T1 depressive and insomnia symptoms were entered to the model explaining 1% additional variance in T2 QOL. In Step 3, T1 *DSM*-5-TR prolonged grief symptoms were added to the model explaining 0% additional variance in T2 QOL.

## Discussion

The aim of the present study was to shed light on the criterion validity of the two similar but distinct versions of PGD included in the ICD-11 and *DSM*-5-TR, by examining associations between both sets of prolonged grief symptoms and QOL concurrently and longitudinally. Descriptive (zero-order) correlational analyses demonstrated that both sets of prolonged grief symptoms (as well as depressive and insomnia symptoms) related negatively to QOL concurrently and longitudinally. Subsequently, we demonstrated that ICD-11 and *DSM*-5-TR prolonged grief symptoms related negatively to QOL concurrently, while controlling for co-occurring depressive and insomnia symptoms. Lastly, we showed that ICD-11 prolonged grief symptoms (but not *DSM*-5-TR prolonged grief symptoms) predicted residual change in QOL at 6-month follow-up, also while controlling for depression and insomnia symptoms. Thus, while both sets of prolonged grief symptoms showed evidence of concurrent validity, only ICD-11 prolonged grief symptoms predicted QOL in the most stringent tests of predictive validity. Put differently, the evidence for the criterion validity of ICD-11 PGD provided by our analyses was consistent, while the evidence for the criterion validity of the *DSM*-5-TR PGD was not.

Our study replicated some past findings using the ICD-11 and *DSM*-5-TR symptom sets of PGD. In line with previous studies (e.g. Maccallum and Bryant, 2020; Silverman et al., 2000), we also found negative concurrent associations between prolonged grief symptoms and QOL, supporting the concurrent validity of these criteria sets.

Relatedly, our zero-order correlation analyses demonstrated longitudinal negative associations between prolonged grief symptoms and QOL, as reported in a rare longitudinal study (Boelen and Prigerson, 2007). However, our more stringent longitudinal analyses (controlling for baseline QOL and, additionally, symptoms of depression and insomnia) uniquely illustrated differences in criterion validity evidence between current prolonged grief symptom sets. That is, ICD-11 prolonged grief symptoms showed small longitudinal effects on QOL, whereas *DSM*-5-TR prolonged grief symptoms did not. Therefore, more consistent evidence was found for the predictive validity of ICD-11 prolonged grief symptoms than for *DSM*-5-TR prolonged grief symptoms. However, it should be noted that the confidence intervals for the regression weights overlapped for all regression models examining associations between ICD-11 and *DSM*-5-TR prolonged grief symptoms and QOL (Cumming, 2009). This could imply that the criterion validity of both symptom sets of PGD may be similar in the population, despite differences in significance of predictive relationships. Therefore, our results lend only partial support for the idea that differences between these new PGD symptom sets (e.g. in symptom count, content and timing criteria) result in different phenomenological characteristics (Eisma et al., 2022; see also: Boelen and Lenferink, 2020; Haneveld et al., 2022).

A first explanation for the diverging results from our controlled longitudinal analyses is the difference in timing criteria between both versions of PGD. Within the present study, we selected bereaved adults based on time since loss (⩾6 months according to ICD-11, ⩾12 months according to *DSM*-5-TR), so that participants in each sample could potentially qualify for the criteria of PGD specified in these diagnostic systems. In a recent multi-wave study applying random-intercept cross-lagged analyses, the effects of grief on depression and posttraumatic stress symptoms were shown to be more pronounced earlier compared with later in the grief process (Komischke-Konnerup et al., 2021). Therefore, ICD-11 prolonged grief symptoms may be a more powerful predictor of QOL than *DSM*-5-TR prolonged grief symptoms, because acute grief reactions show relatively stronger effects on mental health than more protracted grief reactions. This may provide an argument in favor of a shorter post-loss timing criterion for PGD (i.e. 6 instead of 12 months), as this can offer more opportunities to mitigate negative effects of severe grief earlier in the grief process. This appears feasible, as illustrated by randomized controlled trials into preventive grief therapy in the acute phase of loss showing significant effects on grief and related mental health problems (e.g. Litz et al., 2014; Reitsma et al., 2023).

A second potential explanation for this pattern of findings is that symptoms unique to ICD-11 PGD (i.e. difficulty accepting the loss, guilt, blame, difficulty experiencing positive mood) may affect general well-being more strongly than symptoms unique to *DSM*-5-TR PGD (e.g. avoidance of loss, feelings of meaninglessness, intense loneliness). Further scrutiny of these associations is warranted. Unraveling these associations may be complex though, as different prolonged grief symptoms also appear to relate to different components of QOL. For instance, a recent network analysis demonstrated that experiencing more meaningless and role confusion related to lower psychological QOL, whereas difficulties trusting others related to lower social QOL (Maccallum and Bryant, 2020). It has yet to be established how current ICD-11 and *DSM*-5-TR prolonged grief symptoms relate to QOL components concurrently and longitudinally. Longitudinal network analyses may form an important step in unraveling these relationships.

### Strengths, limitations and directions for future research

The present study is one of the first to demonstrate both similarities and differences in the criterion validity evidence of ICD-11 and *DSM*-5-TR PGD, with stronger evidence emerging for ICD-11 symptom set. Strengths of this study include a two-wave survey design and the statistical control for related psychopathology symptoms and baseline levels of the criterion QOL in longitudinal analyses. However, some limitations of this study should be acknowledged.

First, our sample consisted primarily of higher educated, middle-aged women from a Western country. Overrepresentation of middle-aged females is common in grief research (Eisma and Stroebe, 2021) and may partly reflect an overrepresentation of older women in widowhood (Arbuckle and de Vries, 1995) as well as a stronger inclination to women to share their feelings (Stroebe et al., 2001). While there are no reasons to assume different outcomes in bereaved samples with lower education levels, more men, and people from different cultural backgrounds, the generalizability of current findings remains to be established. Second, since we selected our samples based on time since loss, the sample in which we examined the effects of *DSM*-5-TR prolonged grief symptoms on QOL (Sample 2) was smaller than the sample used to examine effects of ICD-11 prolonged grief symptoms on QOL (Sample 1). While this can negatively affect statistical power, the effects of *DSM*-5-TR prolonged grief symptoms on QOL in our most stringent longitudinal analyses were negligible in size (i.e. approximately 0% explained variance). Such effects would thus have been unlikely to be detected in a sample of similar in size as Sample 1. Third, prolonged grief symptom levels ranged from nonclinical to clinical in our samples and we did not perform clinical interviews, and as such could not establish diagnoses of ICD-11 and *DSM*-5-TR PGD. Examining QOL between people with and without these disorders remains an important goal for future research. Fourth, we only examined the performance of both ICD-11 and *DSM*-5-TR prolonged grief symptoms in relation to a single criterion, QOL. Future research may use multiple criteria, including subtypes of QOL and other indicators of well-being and mental and physical health.

## Conclusion

Despite these limitations, the present study is one of the first to illustrate differences in associations of ICD-11 prolonged grief symptoms with a key criterion of general well-being compared with *DSM*-5-TR prolonged grief symptoms. Our findings suggest that prolonged grief symptoms per ICD-11 may have similar concurrent validity as prolonged grief symptoms per *DSM*-5-TR, yet a better predictive validity. This study aligns with past research demonstrating differences in the phenological characteristics of both new PGD criteria sets (e.g. Boelen and Lenferink, 2020; Haneveld et al., 2022). Thereby, it forms a stepping-stone in establishing when these criteria sets behave similarly or differently, which provides indications of how criteria sets may be refined in future versions of pathological grief in diagnostic handbooks.

## Supplemental Material

sj-docx-1-anp-10.1177_00048674241249601 – Supplemental material for ICD-11 and DSM-5-TR prolonged grief symptoms and quality of life: A criterion validity test Supplemental material, sj-docx-1-anp-10.1177_00048674241249601 for ICD-11 and DSM-5-TR prolonged grief symptoms and quality of life: A criterion validity test by Maarten C Eisma and Lara O Schmitt in Australian & New Zealand Journal of Psychiatry
