# Supplementary material for: ICD-11 and DSM-5-TR prolonged grief symptoms and quality of life: A criterion validity test
Source: Aust N Z J Psychiatry. 2024 May 6;58(8):693–701. doi: 10.1177/00048674241249601 (PMC11308347; doi:10.1177/00048674241249601)
Supplement: sj-docx-1-anp-10.1177_00048674241249601 – Supplemental material for ICD-11 and DSM-5-TR prolonged grief symptoms and quality of life: A criterion validity test [file sj-docx-1-anp-10.1177_00048674241249601.docx]

**Supplemental Table A**

*Group Comparison of Participants in Sample 2 vs. not in Sample 2*

| Demographic Variables | | Sample 2  (*N =* 210) | Not in Sample 2  (*N* = 197) | Group Comparisons |
| --- | --- | --- | --- | --- |
| Sex, *n* (%) | |  |  |  |
|  | Woman | 186 (89) | 174 (88) | χ^2^(1, *N*  = 407) = .006, *p* =.938 |
| Age in years, *M* (*SD*) | | 53.66 (13.47) | 52.62 (11.91) | 𝑈 = 21948.5, *p* = .287 |
| Level of education ^a^, *n* (%) | |  |  |  |
|  | Higher education | 121 (58) | 114 (58) | χ^2^(1, *N* = 407) = .003, *p* = .959 |
|  | Lower education | 89 (42) | 83 (42) |  |
| The deceased was, *n* (%) | |  |  | χ^2^(4, *N* = 407) = 14.393, *p* = .006 |
|  | Partner | 112 (53) | 81 (41) | χ^2^(1, *N* = 407) = 6.084, *p* = .014 |
|  | Parent | 47 (22) | 78 (40) | χ^2^(1, *N* = 407) = 14.152, *p* < .001 |
|  | Sibling | 14 (7) | 9 (5) | χ^2^ (1, *N* = 407) =.839, *p* = .360 |
|  | Child | 27 (13) | 22 (11) | χ^2^(1, *N* = 407) = .274, *p* = .601 |
|  | Other persons | 10 (5) | 7 (4) | χ^2^(1, *N* = 407) = .371, *p* = .542 |
| Time since the loss in months, *M* (*SD*) | | 30.05 (17.75) | 8.21 (1.32) | 𝑈 = 41370, *p* < .001 |
| Cause of death^b^, *n* (%) | |  |  |  |
|  | Nonviolent | 171 (81) | 178 (90) | χ^2^(1, *N* = 407) = 6.628, *p* = .010 |
|  | Violent | 39 (19) | 19 (10) |  |
| Sex of the deceased, *n* (%) | |  |  |  |
|  | Man | 155 (74) | 119 (60) | χ^2^(1, *N* = 407) = 8.301, *p* = .004 |
| The loss was, *n* (%) | |  |  | χ^2^(2, *N* = 407) = 1.549, *p* = .461 |
|  | Expected | 68 (32) | 68 (35) |  |
|  | Unexpected | 111 (53) | 93 (47) |  |
|  | Both or neither | 31 (15) | 36 (18) |  |

*Note*. Sample 2 includes participants whose loss occurred ≥ 12 month ago; Not in sample 2 includes participants whose loss occurred ≥ 6 months and < 12 months ago. ^a^Higher education = college and university, Lower education = education levels lower than college or university; ^b^ Nonviolent loss = natural deaths and coronavirus deaths, Violent loss = accident or suicide.
